# Effects of a protection gradient on carnivore density and survival: an example with leopards in the Luangwa valley, Zambia

**DOI:** 10.1002/ece3.2155

**Published:** 2016-05-05

**Authors:** Elias Rosenblatt, Scott Creel, Matthew S. Becker, Johnathan Merkle, Henry Mwape, Paul Schuette, Twakundine Simpamba

**Affiliations:** ^1^ Zambian Carnivore Programme PO Box 80 Mfuwe Eastern Province Zambia; ^2^ Department of Ecology Montana State University Bozeman Montana 59717; ^3^ University of Alaska Anchorage Alaska Center for Conservation Science 3211 Providence Drive Anchorage Alaska 99508; ^4^ Department of National Parks and Wildlife Private Bag 1 Kafue Road Chilanga Zambia

**Keywords:** Anthropogenic effects, bushmeat, harvest, leopard, *Panthera pardus*, prey depletion, robust design

## Abstract

Human activities on the periphery of protected areas can limit carnivore populations, but measurements of the strength of such effects are limited, largely due to difficulties of obtaining precise data on population density and survival. We measured how density and survival rates of a previously unstudied leopard population varied across a gradient of protection and evaluated which anthropogenic activities accounted for observed patterns. Insights into this generalist's response to human encroachment are likely to identify limiting factors for other sympatric carnivore species. Motion‐sensitive cameras were deployed systematically in adjacent, similarly sized, and ecologically similar study areas inside and outside Zambia's South Luangwa National Park (SLNP) from 2012 to 2014. The sites differed primarily in the degree of human impacts: SLNP is strictly protected, but the adjacent area was subject to human encroachment and bushmeat poaching throughout the study, and trophy hunting of leopards prior to 2012. We used photographic capture histories with robust design capture–recapture models to estimate population size and sex‐specific survival rates for the two areas. Leopard density within SLNP was 67% greater than in the adjacent area, but annual survival rates and sex ratios did not detectably differ between the sites. Prior research indicated that wire‐snare occurrence was 5.2 times greater in the areas adjacent to the park. These results suggest that the low density of leopards on the periphery of SLNP is better explained by prey depletion, rather than by direct anthropogenic mortality. Long‐term spatial data from concurrent lion studies suggested that interspecific competition did not produce the observed patterns. Large carnivore populations are often limited by human activities, but science‐based management policies depend on methods to rigorously and quantitatively assess threats to populations of concern. Using noninvasive robust design capture–recapture methods, we systematically assessed leopard density and survival across a protection gradient and identified bushmeat poaching as the likely limiting factor. This approach is of broad value to evaluate the impacts of anthropogenic activities on carnivore populations that are distributed across gradients of protection.

## Introduction

Large carnivore populations are declining globally, often due to anthropogenic effects (Vitousek et al. 1997; Ripple et al. 2014), despite their ecological, economic, and social importance (Estes et al. 2011; Ripple et al. 2014). These species are difficult to study due to their cryptic and often solitary nature, requiring substantial effort to accurately describe their status and viability (Durant et al. 2007). Most carnivore populations reside within and adjacent to protected areas (PAs), which face increasing pressure from rapid human population growth on their periphery (Wittemyer et al. 2008). Peripheral human encroachment and activities can limit carnivore populations within PAs (edge effects; Pulliam 1988; Woodroffe and Ginsberg 1998) and can draw individuals from PAs (attractive sinks; Loveridge et al. 2010). To lessen edge effects and illegal activity within PAs, buffer zones are often established, or fences constructed, but the efficacy of these measures has recently been questioned (Geldmann et al. 2013; Lindsey et al. 2014; Durant et al. 2015). With growing human populations, there is a clear need to identify how gradients of protection from anthropogenic activities affect carnivore populations, to inform wildlife management and land‐use planning.

**Figure 1:**
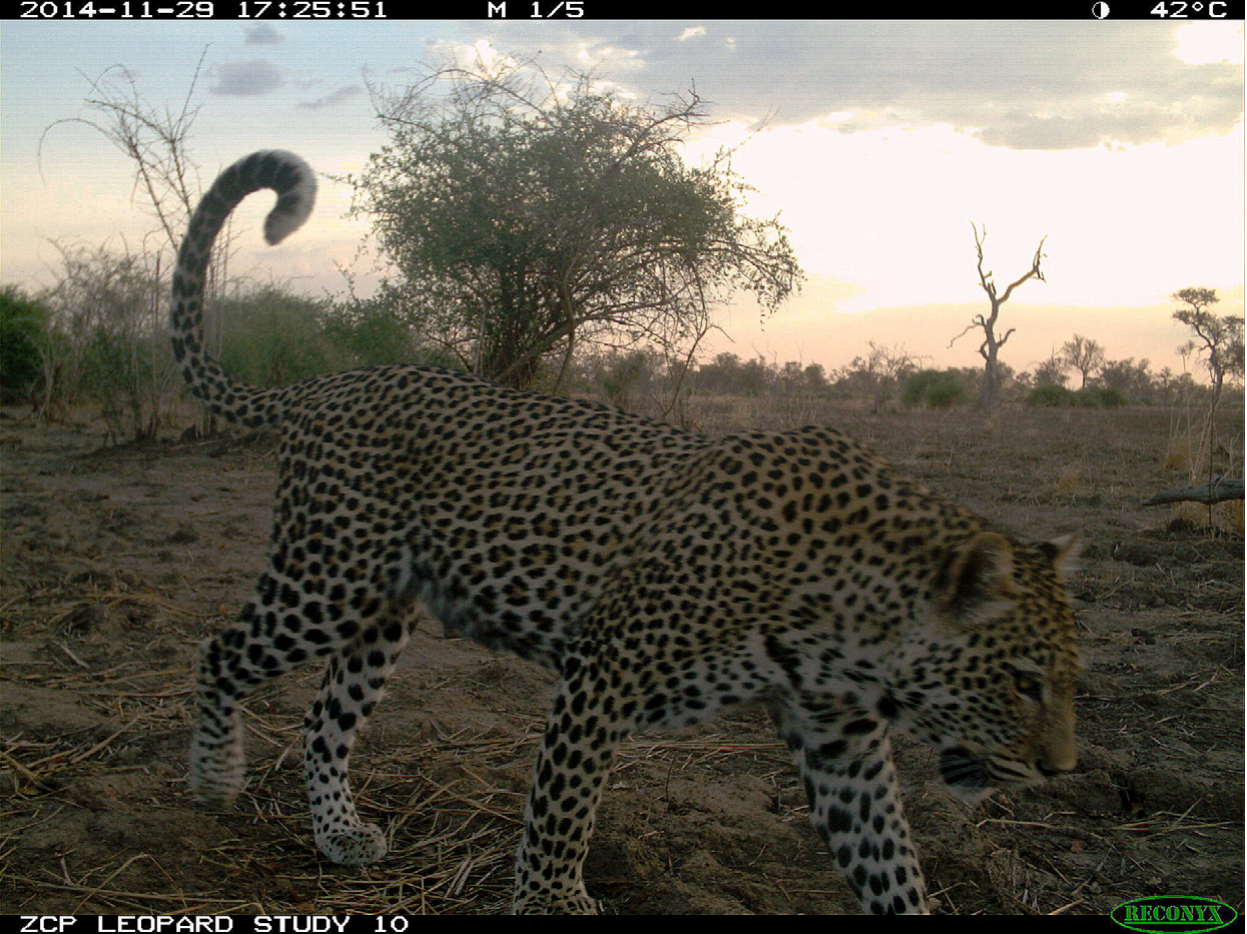
A leopard photographed by a remote camera trap traveling in the late afternoon in South Luangwa National Park, Zambia. Photograph by E. Rosenblatt

Leopards (*Panthera pardus*; Fig. 1) are broadly distributed throughout Africa and Asia in ecosystems with a wide array of prey and habitats (Hayward et al. 2006; Henschel et al. 2008). Despite the species' generalist ecology, recent studies indicate range contraction due to human encroachment, poorly regulated harvest, poaching, and conflict (Henschel et al. 2008). Zambia contains regionally significant leopard populations (Purchase et al. 2007) in a complex of national parks and adjacent Game Management Area buffer zones (GMAs; Lewis and Alpert 1997). Most of these populations have received little study (Ray 2011) and face rapid human population growth, encroachment, and wire snaring for bushmeat. These anthropogenic pressures have reduced the ecological functionality of GMA buffer zones and are thought to directly and indirectly affect carnivore populations through snaring by‐catch, prey depletion, and habitat loss (Watson et al. 2013, 2014; Lindsey et al. 2014). The effects of anthropogenic activities on protected leopard populations have been investigated in several ecosystems across their distribution (Balme et al. 2009b, 2010; Henschel et al. 2011; Swanepoel et al. 2015), but the status of most leopard populations remains unknown, while anthropogenic pressures around them are intensifying. Therefore, improving our understanding of leopards' responses to anthropogenic pressures is critical for their conservation and will likely indicate limiting factors for other sympatric, unstudied carnivore populations facing the same anthropogenic pressures. Developing methods that address these questions efficiently is a high priority.

South Luangwa National Park (SLNP) is Zambia's premiere photograph‐tourism destination, and the GMAs adjacent to SLNP are categorized by the Zambian Department of National Parks and Wildlife as “prime” for trophy hunting. The Luangwa Valley, which encompasses SLNP, contains what is thought to be Zambia's largest leopard population, but there are limited data to support this assertion (Ray 2011). Although most of SLNP is surrounded by GMAs, human encroachment is increasing with demonstrated effects on other carnivores. The drivers of these effects include illegal bushmeat harvest (Lewis and Phiri 1998; Becker et al. 2013a; Lindsey et al. 2013; Watson et al. 2013), high levels of legal trophy and resident hunting (Yamazaki 1996; Becker et al. 2013b; Rosenblatt et al. 2014), and habitat conversion (Watson et al. 2014). Therefore, SLNP and adjacent GMAs provide an opportunity to compare leopard densities and survival rates between a fully protected area with relatively minimal impact by humans, and an immediately adjacent buffer zone with growing direct and indirect anthropogenic effects. Estimated differences in density or survival areas across this protection gradient can help identify which anthropogenic pressure(s) limits leopards and other sympatric carnivores.

In this study, we use motion‐sensitive cameras and mark–recapture methods to estimate leopard density and survival rates in a portion of the South Luangwa leopard population from 2012 to 2014. We use these estimates to determine how density and survival change across a gradient of protection, and identify the likely drivers. This study contributes to national and regional efforts to reduce the negative effects of human population growth around PAs, and establishes effective tools to identify anthropogenic threats and guide large carnivore management.

## Materials and Methods

### Study area

**Figure 2:**
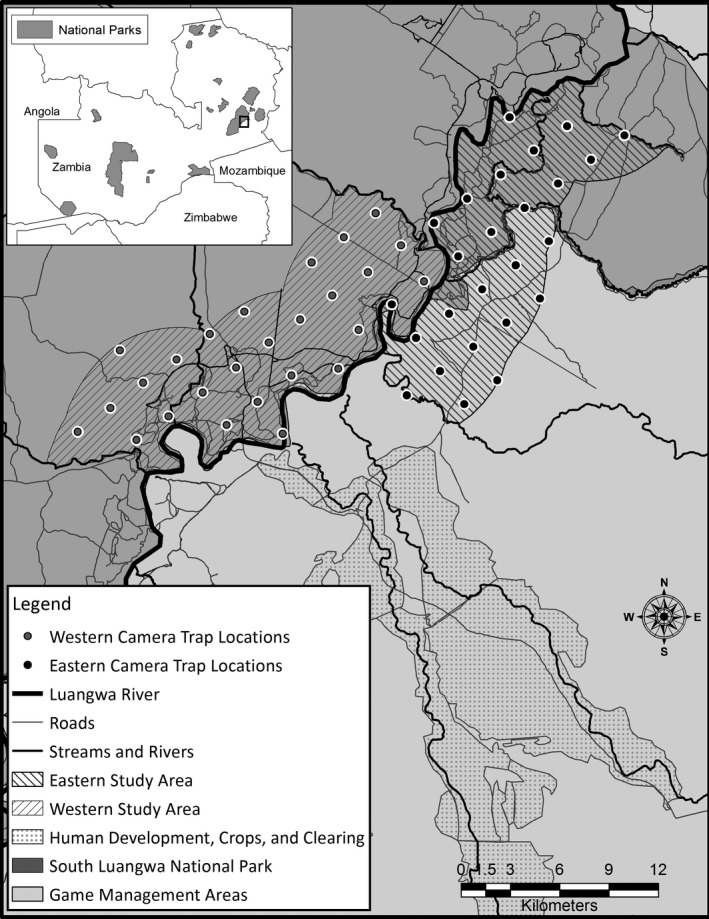
Our two study areas spanned the border of South Luangwa National Park, Eastern Province, Zambia. Camera traps surveyed strictly protected areas (western study area – WSA) and community game management areas (eastern study area – ESA) encompassing a gradient of management regimes and accompanying human impacts likely to influence density and survival.

Our study was conducted in two adjoining areas on the boundary of SLNP (S13.07958 E31.77407; 9050 km^2^) and Lupande GMA (5660 km^2^), allowing comparison of leopard densities across management regimes differing in the degree and type of human activity (Fig. 2). SLNP is strictly protected as an IUCN Category II Protected Area, with photographic safari tourism and law enforcement patrols, although some illegal wire‐snare and rifle poaching does occur. GMAs are IUCN Category VI areas intended as buffer zones allowing a variety of natural resource‐based uses (Chomba et al. 2011). Human settlements are permitted and increasing in the Lupande GMA (and most GMAs in Zambia), causing an array of conservation concerns (Lewis and Phiri 1998; Becker et al. 2013a; Watson et al. 2013, 2014). Legal trophy hunting of adult male leopards, other large carnivores and herbivores occurred in Lupande and other GMAs, except during a January 2013–April 2015 moratorium. Livestock densities were locally low, making human–carnivore conflict uncommon relative to other studies (e.g., Marker and Dickman 2005).

The Luangwa River forms most of the eastern border of SLNP and the western border of Lupande GMA. As the largest perennial water source in the region, wildlife and human activity is centered along the river boundaries of SLNP and adjacent GMAs, particularly during the dry season (May–November). Large mammals move across the Luangwa River freely, particularly in the late dry season (Rosenblatt et al. 2014); thus, we considered our two study areas as segments of the same leopard population, which we termed the western study area (WSA) and eastern study area (ESA) (Fig. 2). The WSA (172 km^2^) is located in SLNP on the western side of the Luangwa River ranging between the seasonal Katete and Luwi Rivers and includes areas within 6 km of the Luangwa River. The ESA (141 km^2^) is located on the eastern bank of the Luangwa River, bounded by the seasonal Mwangazi and spring‐fed Chichele streams and also includes areas within 6 km of the Luangwa River. The ESA includes portions of the Lupande GMA and the Nsefu sector of SLNP, a small portion of the park situated on the eastern side of the Luangwa River. Other than differences in human use, the ESA and WSA were selected to be ecologically similar, with comparable compositions of edaphic grassland, deciduous riparian forest, miombo (*Brachystegia spp*) woodland, mopane (*Colophospermum mopane*) woodland and scrubland, dry deciduous forest and undifferentiated woodland (Astle et al. 1969; White 1983; Astle 1988). Our sampling on the ESA and WSA was designed to provide strong inferences in the following ways: (1) The two sites were selected to be similar for variables (other than those directly related to the level of protection) that would be expected to influence leopard density or demography (e.g., vegetation type and proximity to permanent water). (2) The two sites were of the same size, were spatially close, and were sampled over highly overlapping time periods. (3) Within primary sampling periods, the sampling design for both sites was identical.

### Study design

We used a systematic camera grid to photograph leopards within each study area and used closed robust design capture–recapture models to estimate population size and annual survival rates (Pollock 1982; Kendall et al. 1995). In both study areas, we placed cameras using a square grid that was random in its origin and orientation. Spacing for this grid followed established procedures for large felids to meet the assumptions of closed mark–recapture models (Otis et al. 1978; Karanth and Nichols 1998; Balme et al. 2009a). We based grid cell size on the smallest home range estimate available (14 km^2^) for an adult female leopard in Zambia's Luambe National Park (approximately 60 km from our study; Ray 2011), and spaced trap sites 2.5 km from each other (Fig. 2). This spacing was intended to place multiple trap sites within the home range of each individual (Karanth and Nichols 1998).

We established 25 and 26 unbaited camera‐trap sites (hereafter sites) in the ESA and WSA, respectively (Fig. 2). We selected sites by searching for leopard tracks within 100 m of each grid point (Silver et al. 2004). If we encountered no tracks, we selected the most active game trail within 100 m of the point. Cameras were attached to trees at a height and angle intended to maximize the likelihood of being triggered by leopards. One site was located more than 100 m from the grid point, because no trees were available within the 100 m radius. Vegetation varied between sites but all were in vegetation types used by leopards (Balme et al. 2007). At each site we set two Reconyx Hyperfire PC800 cameras (Reconyx, Inc., Holmen, WI) facing each other to photograph both sides of passing leopards, set to take five photographs in succession upon detection of movement. We visited sites on foot in small groups to minimize our potential impact on subsequent detections. We downloaded photographs when cameras were moved between locations (see below). We identified individuals using spot patterns and sexed them using genitalia and sexually dimorphic traits such as body and head size and the prominence of their neck dewlap (Balme et al. 2012). We did not assign ages due to the limitations of image quality and the difficulty of aging leopards accurately (Balme et al. 2012). We created capture histories for each individual denoting detections (1) and nondetections (0) on each day of camera trapping.

### Robust design model selection

We used an extended robust design model to estimate population size (N) for each study area, annual survival (*S*), detection and redetection probabilities (*p* and *c* respectively), and rates of temporary emigration (*γ″*, the probability of an individual temporarily moving off of the study area and becoming unavailable for capture and *γ*′, the probability of an individual remaining outside of the study area and thus remaining unavailable for capture). We hypothesized that density would vary by study area and annual survival rates would vary by gender, study area, and time, with a potential interaction between gender and study area. We compared models with random, Markovian, or no temporary emigration (Kendall et al. 1997). Finally, we tested for effects on detection probability of study area and season (see below) and whether detection probability differed from redetection probability to evaluate whether our activity at trap sites was impacted leopard behavior.

For each primary sampling occasion on each study area, we estimated population size during an 87‐day period, a period we selected a priori to satisfy the assumption of population closure based on other large felid camera‐trap studies (Karanth and Nichols 1998). Each of these primary sampling occasions fell entirely within the cold dry season (CD, May–August) or the hot dry season (HD, September–November), so we refer to primary sampling occasions as seasons hereafter. Wet season data collection was not possible because portions of both study areas were inaccessible. Because we had too few cameras to survey all locations simultaneously, we deployed camera traps in a random rotation across four “sections” within each study area (Karanth 1995). Each section consisted of 6–7 sites and was sampled for 21 days (21 days/section X four sections + 3 days to redeploy cameras = 87 days). We created encounter histories for each individual pooled across sections for a 21‐day period for each study area in each season. We broke each primary occasion into three 7‐day secondary occasions a priori to maximize detection probability.

In total, five seasons were surveyed over 3 years in the WSA (CD 2012, CD 2013, HD 2013, CD 2014, and HD 2014) and four seasons were surveyed over 2 years in the ESA (CD 2013, HD 2013, CD 2014, and HD 2014). With the rotation design to maximize spatial replication, there were staggered periods of 69–70 days between CD and HD samplings. We considered these periods as open to contribute to survival estimates, in addition to the open periods during wet seasons between HD and CD sampling (CD 2012 – CD 2013 = 349 days, HD2013 ‐ CD2014 = 262 days). One ESA section was not sampled in the HD season of 2013 due to early rains, so our estimates for this season are based on 76% of the camera‐days used for estimate in the other seasons. Survival rates are expressed as annual rates, exponentiating as needed to account for the time over which survival was estimated.

The robust design model includes several parameters (*p, c, γ*′, and *γ*”) that must be estimated to provide unbiased estimates of population size and survival rates, but were not of direct interest in this study. To focus on the parameters of interest, we evaluated models in two steps, using Akaike's information criteria corrected for small sample size and overdispersion (QAICc) in the RMark package of R (Laake 2013). To correct for overdispersion, we estimated a median $\hat{c}$ value by collapsing the secondary sessions within each season and fitting a time‐varying Cormack–Jolly–Seber model to the data, as suggested by J. Laake (pers. commun.). All confidence intervals were then corrected for overdispersion in survival rates using this median $\hat{c}$ value as a variance inflation factor. In the first step of model selection, we identified the best model of annual survival out of 10 candidate models (Table 1) with a single estimate of detection probability $\hat{p}\left(.\right)$ and no temporary emigration (*γ″* = 0 and *γ*′ = 1). The model(s) receiving the majority of the QAICc weight was selected as the most likely parameterization for annual survival. In the second step, we used the best model(s) for annual survival to test our hypotheses for each of the remaining parameters, resulting in a set of 72 candidate models. In fitting these 72 candidate models, we eliminated any that showed signs of overparametrization. We identified the top models of remaining candidate models (*n* = 32; Table A1) using QAICc and used model averaging (with the *collect.modelsl()* and *model.average()* functions of the RMark package) to estimate parameter values across sex, study area, and time. We calculated overdispersion‐corrected 95% confidence intervals for parameters in each study area using model‐averaged seasonal estimates with pooled variances.

**Table 1:** Model selection results using QAICc to determine the best‐supported robust design model of survival (*S*): In the text, this is step one of model selection. Models varied only by their parameterization of *S*. In all models, there was no temporary emigration (*γ″*=0, *γ*′=1) and a single detection probability (*p*(.)), and population size was estimated by season and study area (*N*). From these results, we used the top three parameterizations of *S* for the second stage of model selection

Model	Parameters	Delta QAICc	QAICc weight
S(.),*γ″*(0), *γ*′(1),p(.),N(season+area)	8	0.00	0.50
S(sex), *γ″*(0), *γ*′(1),p(.),N(season+area)	9	1.61	0.22
S(area), *γ″*(0), *γ*′(1),p(.),N(season+area)	9	2.37	0.15
S(sex+area), *γ″*(0), *γ*′(1),p(.),N(season+area)	10	3.99	0.07
S(area*sex), *γ″*(0), *γ*′(1),p(.),N(season+area)	11	6.44	0.02
S(time), *γ″*(0), *γ*′(1),p(.),N(season+area)	11	6.94	0.02
S(time+sex), *γ″*(0), *γ*′(1),p(.),N(season+area)	12	8.56	0.01
S(area+time), *γ″*(0) *γ*′(1),p(.),N(season+area)	12	9.34	0.00
S(time+sex+area), *γ″*(0), *γ*′(1),p(.),N(season+area)	13	11.00	0.00
S(time+area*sex), *γ″*(0), *γ*′(1),p(.),N(season+area)	14	13.62	0.00

We estimated density for each study area by dividing population estimates by the area surveyed. We estimated this area by calculating the mean maximum distance moved (MMDM; Stickel 1954; Wilson and Anderson 1985) across all individuals from both study areas and buffering each trap site by half of the MMDM distance (HMMDM). Balme et al. (2009a) found that without telemetry data, using HMMDM and buffering each camera‐trap site (Silver et al. 2004) was the least biased estimator for leopard density when compared to independent estimates of density from intensive telemetry data. Some recent research questions whether MMDM or HMMDM compares more closely to density estimates derived from telemetry data and spatially explicit capture–recapture models (SECR; Efford 2004) in large felids, and that HMMDM may overestimate density estimates by underestimating space use of individuals (e.g., Tobler and Powell 2013). We chose to follow Balme et al. (2009a)'s leopard‐specific recommendation for density estimates, but we provide density estimates based on average MMDM measures in the Appendix (Table A2). A strength of our study design is that the choice of HMMDM vs. MMDM has no effect on differences in density between the ESA and WSA (the primary interest of this study). In this study, the choice is relevant only for comparisons of our density estimates to those from other studies. We did not implement spatially explicit capture–recapture models (SECR; Efford 2004) because current implementations require assumptions about space‐use distributions that are not likely to be met by leopards, and our sampling was carefully designed to limit differences in the area sampled for the two sites. By sampling in immediately adjacent areas with identical sampling grids and methods, we minimized problems related to estimation of sampling area that can arise during conversion of population size to population density, thus avoiding the primary problem that SECR attempts to address.

## Results

**Figure 3:**
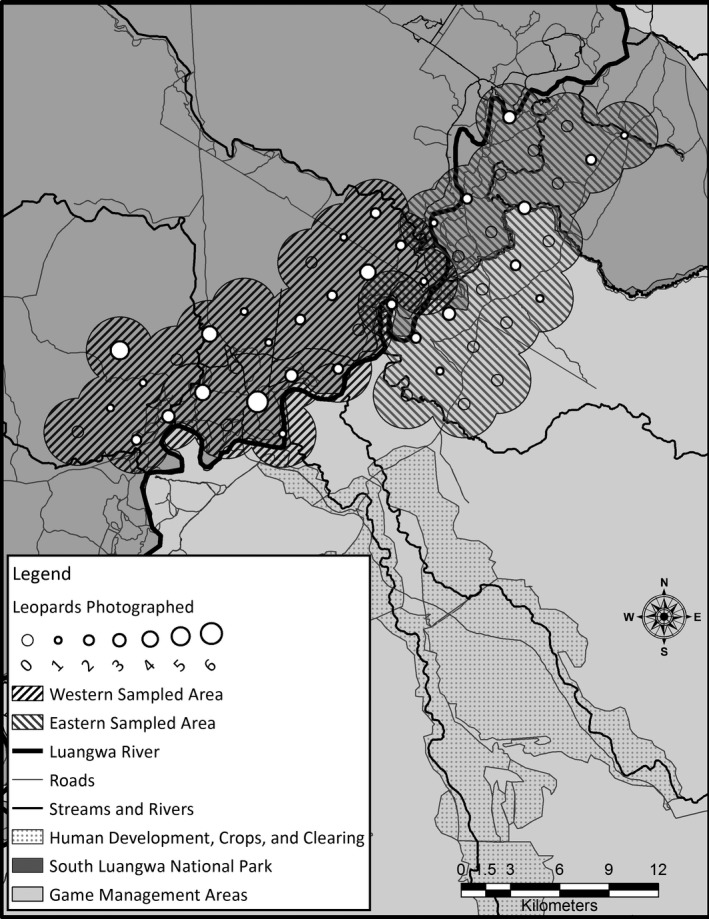
The distribution of leopard detections across the ESA and WSA. The size of the circles indicates the number of individual leopards that were detected at each camera‐trap site. The shaded polygons indicate each study area's trap‐buffer, that is, the area effectively sampled for the calculation of density.

We photographed 51 leopards over the 3 year study, from 8730 successful camera‐trap days. Of these 51 leopards, twenty leopards were photographed only from one side due to photograph angles and camera failure or theft. To avoid double‐counting individual leopards, we created encounter histories using only right‐sided photographs, resulting in 43 individually recognized leopards used in this analysis. We documented 28 individuals in WSA and 15 individuals in ESA on 99 occasions, with 26 individuals photographed on more than one occasion. No individuals were detected in both study areas. We observed male:female ratios of 1:1.5 and 1:1.8 in the ESA and WSA, respectively (1:1.7 overall). Five leopards were not sexed on the basis of genitalia and were designated as females for the analysis based on their size and neck characteristics (Balme et al. 2012). In the WSA, leopards were photographed at 77% of the trap sites, with most leopards detected along seasonal streams and the Luangwa River (Fig. 3). In the ESA, leopards were photographed at 48% of trap sites, most frequently along the Luangwa River (Fig. 3).

Using QAICc scores (median $\hat{c}$ = 1.92) to compare models with constant detection and no temporary emigration (Table 1), the best‐supported model of survival (*S*) had a single mean rate for all individuals, but appreciable QAICc weight also went to models with a difference between the sexes (slightly lower survival in males) and with a difference between sites (slightly lower survival within the ESA). When we varied the parameterizations of temporary emigration and detection probability, QAICc scores identified 10 models within four units of the best‐supported model (Table 2). Model‐averaged survival estimates were higher for females and did not differ by area (Table 3), but confidence intervals on these estimates were broad compared with observed differences. Model‐averaged estimates indicated very low levels of temporary emigration, with precision insufficient to determine whether movements were Markovian or random (Table 3). The model‐averaged detection probability estimate was higher in the WSA than the ESA, but this difference was small relative to the confidence intervals (Table 3). Model‐averaged estimates of detection (*p*) and redetection (*c*) probabilities were nearly equal, confirming that unbaited cameras did not influence recapture probability.

**Table 2:** The best‐supported robust design models from 72 candidate models, as determined by QAICc scores: In the text, this is step two of model selection. In addition to the three best parameterizations of *S*, these top models supported nonexistent (*γ″*(0), *γ*′(1)) or random (*γ″*(.)=*γ*′) temporary emigration and *p* and *c* to be equal and constant (*p*(.)), unequal and constant (*p*(.), c(.)), or equal and differing by area (*p*(area)). These models were used for model‐averaged estimates of *S*,* γ″*,* γ*′, *p*,* c*, and *N*

Model	Parameters	Delta QAICc	QAICc weight
S(.), *γ″*(0), *γ*′(1), p(.), N(season+area)	8	0.00	0.19
S(.), *γ″*(0), *γ*′(1), p(area), N(season+area)	9	0.94	0.12
S(sex), *γ″*(0), *γ*′(1), p(.), N(season+area)	9	1.61	0.08
S(.),*γ″*(.)=*γ*′, p(.), N(season+area)	9	1.93	0.07
S(area), *γ″*(0), *γ*′(1), p(.), N(season+area)	9	2.37	0.06
S(.), *γ″*(0), *γ*′(1), p(.), c(.), N(season+area)	9	2.42	0.06
S(sex), *γ″*(.)=*γ*′, p(area), N(season+area)	10	2.71	0.05
S(.), *γ″*(.)=*γ*′, p(area), N(season+area)	10	3.02	0.04
S(area),*γ″*(0), *γ*′(1), p(area), N(season+area)	10	3.19	0.04
S(.),*γ″*(.)=*γ*′, p(.), c(.), N(season+area)	10	3.33	0.03
S(sex), *γ″*(.)=*γ*′, p(.), N(season+area)	10	3.60	0.03

**Table 3:** Model‐averaged parameter estimates of survival (*S*), temporary emigration (*γ″*and *γ*′), detection (*p*), and redetection (c) probabilities for the South Luangwa leopard population

Parameter	Estimate	SE	95% LCL‐UCL
$\hat{S}$ ‐ male, ESA	0.68	0.24	0.20–0.95
$\hat{S}$ ‐ female, ESA	0.73	0.21	0.25–0.96
$\hat{S}$ ‐ male, WSA	0.68	0.18	0.30–0.91
$\hat{S}$ ‐ female, WSA	0.73	0.14	0.40–0.91
$\hat{\gamma^{\prime }}$	0.81	0.35	0.04–1.00
$\hat{\gamma^{\prime \prime }}$	0.05	0.14	0.00–0.96
$\hat{p}$‐ ESA	0.22	0.08	0.10–0.41
$\hat{p}$ ‐ WSA	0.25	0.07	0.15–0.40
$\hat{c}$‐ ESA	0.21	0.07	0.10–0.38
$\hat{c}$‐ WSA	0.25	0.06	0.15–0.37

The overall mean WSA population estimate (18.66 leopards; 95% CI: 16.50–20.81) was 78% larger than the overall mean ESA population estimate (10.50 leopards; 95% CI: 7.90–13.10), with nonoverlapping 95% confidence limits for the two areas (Table 4). The HMMDM for 14 leopards recaptured at multiple sites was 2.04 km (range: 1.22–6.25 km). We used this HMMDM buffer to estimate the effectively sampled areas as 219.51 and 206.48 km^2^ for WSA and ESA, respectively. Thus, overall mean leopard density was 67% higher in the WSA (8.50 leopards/100 km^2^; 95% CI = 7.52–9.48) relative to the ESA (5.08 leopards/100 km^2^; 95% CI = 3.83–6.34; Table 4). Population size estimates varied seasonally in both study areas, but did not show systematic increase/decline or seasonal changes in local density (Table 4).

**Table 4:** Model‐averaged estimates of seasonal and overall average population size ($\hat{N}$) and density (leopards per 100 km^2^) calculated using HMMDM. There was no apparent trend across cold dry (CD) and hot dry (HD) seasons on population estimates within each study area. $\hat{N}$ for HD 2013 and CD 2014 in both study areas were identical due to the same number of individuals captured. On the ESA in HD 2013, 24% of trap sites could not be sampled due to early onset of the rainy season

Season	Number Captured	$\hat{N}$	SE	95% LCL‐UCL	Density	95% LCL‐UCL Density
CD 2013, ESA	8	12.53	5.35	8.45–53.85	6.07	4.09–26.08
HD 2013, ESA	5	8.67	4.37	5.36–42.72	4.20	2.59–20.69
CD 2014, ESA	5	8.67	4.37	5.36–42.72	4.20	2.59–20.69
HD 2014, ESA	7	12.11	5.78	7.56–53.91	5.87	3.66–26.11
CD 2012, WSA	16	22.29	5.91	17.00–55.65	10.15	7.74–25.35
CD 2013, WSA	10	18.21	5.88	12.02–43.39	8.30	5.48–19.77
HD 2013, WSA	9	15.71	5.20	10.47–39.66	7.16	4.77–18.07
CD 2014, WSA	9	15.71	5.20	10.47–39.66	7.16	4.77–18.07
HD 2014, WSA	12	21.35	6.44	14.42–48.04	9.72	6.57–21.88
Average, ESA	–	10.50	1.33	7.90–13.10	5.08	3.83–6.34
Average, WSA	–	18.66	1.10	16.50–20.81	8.50	7.52–9.48

## Discussion

Large carnivores throughout Africa are rarely studied in a manner that yields data on population density or demography that is sufficiently precise to facilitate science‐based management. Improving our understanding of how leopard populations respond to growing peripheral human activities is important for leopard conservation, but may also help identify factors that limit sympatric carnivore populations. In this study, we detected 67% higher density within the National Park (WSA) relative to the bordering areas (ESA), demonstrating that leopard density responds very strongly to management regimes. Our results align with other studies that have found a negative relationship between human encroachment around PAs and leopard population density (Marker and Dickman 2005; Balme et al. 2010; Henschel et al. 2011; but see Stein et al. 2011). Despite large differences in density, we found little evidence that survival differed between study areas and that temporary emigration was occurring at low levels (although estimates for these last two parameters had low precision, likely due to the difficulty of sampling a large number of individual leopards, and inability to estimate age from camera‐trap photographs, largely taken at night). While these limitations must be recognized, these are the first methodologically and statistically rigorous estimates of leopard density and survival in a population considered critical for regional carnivore conservation. These data identify differences in leopard density between areas differing in management and anthropogenic effects and help to identify factors limiting carnivore populations (see below).

### Determining abundance in comparable study areas

Estimating population density is a common objective for camera‐trap studies, but the best methods are debated (Foster and Harmsen 2012). A central problem lies in estimating the area that has been surveyed, because some detected animals are likely to move beyond the perimeter of a camera‐trap array. The width of the average home range of the target species is often used to estimate this area (Dice 1938). A common alternative method relies on the mean maximum distance moved (MMDM) across trap sites for all captured animals. Studies have compared density estimates derived from telemetry and MMDM data, but their conclusions are inconsistent (Parmenter et al. 2003; Foster and Harmsen 2012). Spatially explicit capture–recapture models can simultaneously estimate population size and the area surveyed, yielding a direct estimate of density (Efford 2004). Rather than relying on these solutions, our study design reduced the problem of estimating the surveyed area, relative to prior studies that placed camera traps at sites frequently used by the target species (Balme et al. 2009a) or used lures to increase detection probability (Du Preez et al. 2014). Instead, we used two highly similar randomized sampling grids. Using a randomized grid, our sampling was not biased to areas of heavy use, and thus is more likely to produce a representative estimate of density for the sampled area. By comparing samples from two study areas of the same size, using the same sampling grid and design, our primary analysis is not complicated by the conversion of population size to population density (although this issue does affect comparison of our estimates to other studies). The application of our design hinges on retaining adequate detection probability for the desired analysis, which is a limitation for wide‐ranging felids, but has the advantage of providing estimates of population density that can be directly compared between surveyed areas and that are representative of those areas.

### Drivers behind observed differences in leopard densities

Density differences between the WSA and the ESA could stem from three limiting factors. First, prey populations in the ESA could be depleted due to ongoing illegal poaching and past legal harvests. Second, the ESA leopard population density could be lower due to direct leopard mortality from poaching or residual effects from trophy harvests prior to the 2012 moratorium. Finally, interspecific competition with the local lion population could limit this leopard population. Below we consider how these three factors may have contributed to the observed results.

**Figure 4:**
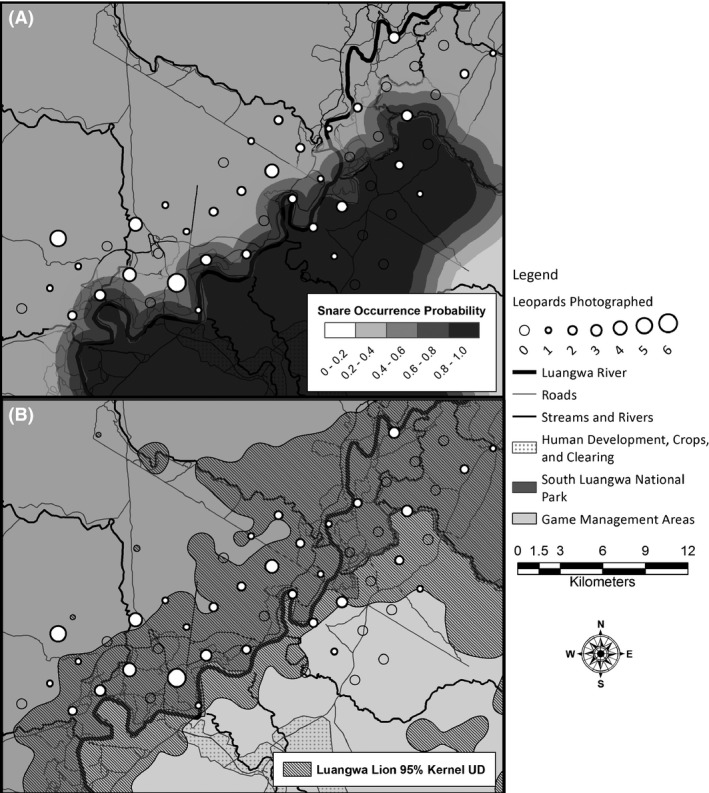
The distribution of leopard encounters compared to (A) gradients of probability of wire‐snare occurrence (from Watson et al. 2013) and (B) patterns of African lion use (95% kernel utilization distribution – from Rosenblatt et al. 2014). Overall, wire‐snare occurrence was higher in the ESA relative to the WSA, and fewer leopards were photographed in areas of high wire‐snare occurrence. Leopards commonly used areas of high lion density and thus do not appear strongly limited by interspecific competition.

Prey depletion provides a coherent explanation for low leopard density in the ESA. A reduction in prey density would be expected to reduce leopard density (Hayward et al. 2007; Balme et al. 2013), and this hypothesis is compatible with the observation that survival rates did not differ between the two areas. Our two study areas differed dramatically in their exposure to wire‐snare poaching. The ESA's median probability of snare occurrence was 5.2‐fold greater than that of the WSA (ESA median= 0.74, range: 0.01–0.99; WSA median=0.14, range:0.01–0.70; Watson et al. 2013; Fig. 4A) due to differences in law enforcement and land use. Herbivore biomass in Lupande GMA was recently estimated as 1/10th of capacity (Lindsey et al. 2014), and in a rough calculation based on interviews in one Lupande GMA community (south of the ESA), Lewis and Phiri (1998) roughly estimated that the community harvested 2 428 animals with wire snares during a 6‐month dry season, the majority of which were impala (*Aepyceros melampus*) and other small‐ and medium‐sized ungulates commonly preyed upon by leopards (Hayward et al. 2006; Ray 2011). Finally, in the Congo Basin, Henschel et al. (2011) documented similar responses of leopard density to exploitative competition with local hunting community. Given the rapid rates of human encroachment for Lupande and the majority of Zambia's GMAs (Watson et al. 2014), illegal bushmeat harvest and consequent prey depletion is probably a strong limiting factor for leopards (and other large carnivores) in GMAs.

Past legal harvests of herbivores may also contribute to prey depletion in GMAs. For decades prior to the 2013–2015 moratorium on trophy and resident hunting, there was active trophy and resident hunting in the ESA for leopard and their prey. Legal hunting did not occur during this study, but prior decades of poorly regulated harvest of herbivores could contribute to prey depletion in the ESA, although legal hunting offtakes were lower than estimated poaching offtake by an order of magnitude. Nevertheless, future management of legal hunting should consider the potentially additive effects of poaching, legal harvest, and rapid rates of habitat loss on prey populations for leopard and other carnivores.

Direct mortality of leopards due to legal or illegal hunting could result in lowered leopard numbers outside of SLNP, as has been demonstrated for the South Luangwa lion population (Rosenblatt et al. 2014), but evidence for this hypothesis is lacking. In the Luangwa valley, wire‐snare poaching is known to be a source of mortality for other large carnivores (Becker et al. 2013a,b; Rosenblatt et al. 2014), but snared leopards are uncommon relative to wild dogs or lions (Rosenblatt unpublished data). Point estimates of survival were lower for males than for females, but this difference was small relative to the width of the confidence intervals, did not differ between the ESA and WSA, and could be due to natural causes. Although legal trophy hunting was not a source of leopard mortality during this study, the effects of trophy hunting on large carnivore density and population structure are likely to persist for some time after the harvest period (Balme et al. 2009b, 2010; Davidson et al. 2011). Leopard quotas in hunting concessions in the Luangwa Valley are high relative to those allocated in the rest of Zambia (Ray 2011), and the ESA included portions of a hunting concession that on average removed 4 male leopards per year. Based on the estimated average population size and the observed sex ratio for the ESA, this rate of offtake would remove most resident males in this study area. Furthermore, male leopards were removed all along the periphery of SLNP, creating the potential for “attractive sink” dynamics (Loveridge et al. 2010) and lowered cub survival due to increased rates of infanticide (Whitman et al. 2004; Balme et al. 2009b, 2010; Packer et al. 2011). Finally given the difficulty in aging leopards (Balme et al. 2012) documented by Ray (2011) in nearby Luambe National Park and the high potential for harvesting female leopards in trophy hunts (Spong et al. 2000), the impacts of past leopard harvests, particularly those that did not adhere to sex‐ or age‐based limits (Balme et al. 2012), are likely to persist for several years. Despite these potential problems, our data provide no evidence that differences in management during the hunting moratorium directly affected leopard mortality rates.

Finally, leopard density can be limited through interspecific competition, mainly with lions (Stander et al. 1997; Balme et al. 2013). If the South Luangwa lion population was the limiting factor for the leopard population, we would expect decreasing leopard densities in areas with recovering lion populations during the hunting ban of 2013–2015. This hypothesis was not supported for two reasons. First, prior to the hunting ban, the WSA was more heavily utilized by lions than the ESA (Fig. 4B), yet after the ban was implemented, the WSA had higher leopard population estimates than the ESA. Second, the frequency of leopard captures across space paralleled the distribution of the local lion population, suggesting that leopards did not avoid areas frequently used by lions in this ecosystem (Fig. 4B). Therefore, there is little evidence that differences in leopard densities were due to interspecific competition with lions.

## Conclusions

The conservation of African large carnivores is increasingly critical given their ecological, economic, and social value, large declines in range and numbers, and the growing threats they face. By quantifying the status and trend of carnivore populations and identifying the factors that control them, we can inform management priorities to mitigate threats. In our study, we detected lower leopard density on the periphery of an important Zambian National Park, likely due to prey depletion driven by bushmeat poaching. If prey depletion limits leopards, it is likely to affect other carnivores, and therefore further research objectives should include rigorous monitoring of both carnivore and herbivore populations across protection gradients. Additionally, with leopard trophy hunting resuming in Zambia in 2015, monitoring should continue to quantify how the density and distribution of leopard respond to altered legal harvest. Our data, combined with this change in policy, provide an ideal opportunity to apply the principles of adaptive management in a manner that is rare for large carnivores. As human encroachment increases adjacent to PAs around the world with strong limiting effects on large carnivores and their prey, research providing reliable and precise estimates of critical population parameters must be in place to evaluate the effectiveness of management decisions.

## Data Accessibility

Leopard capture histories and covariates: uploaded as online supporting information.

## Conflict of Interest

None declared.

## Appendix 1

**Table A1:** The 32 candidate robust design models from the 72 possible candidate models that were not overparameterized. In addition to the three best parameterizations of *S*, these models included nonexistent (*γ″*(0), *γ*′(1)), Markovian (*γ″*(.), *γ*′ (.)), or random (*γ″*(.)=*γ*′) temporary emigration and *p* and *c* to be equal and constant (*p*(.)), unequal and constant (*p*(.), c(.)), equal and differing by area (*p*(area)), unequal and differing by area (*p*(area), c(area)), or equal and differing by season (p(season))

Model	Parameters	Delta QAICc	QAICc weight
S(.), γ″(0), γ′(1), p(.), N(season+area)	8	0.00	0.19
S(.), γ″(0), γ′(1), p(area), N(season+area)	9	0.93	0.12
S(sex), γ″(0), γ′(1), p(.), N(season+area)	9	1.61	0.08
S(.),γ″(.)=γ′, p(.), N(season+area)	9	1.93	0.07
S(area), γ″(0), γ′(1), p(.), N(season+area)	9	2.37	0.06
S(.), γ″(0), γ′(1), p(.), c(.), N(season+area)	9	2.42	0.06
S(sex), γ″(0), γ′(1), p(area), N(season+area)	10	2.71	0.05
S(.), γ″(.)=γ′, p(area), N(season+area)	10	3.02	0.04
S(area),γ″(0), γ′(1), p(area), N(season+area)	10	3.19	0.04
S(.),γ″(.)=γ′, p(.), c(.), N(season+area)	10	3.33	0.03
S(sex), γ″(.)=γ′, p(.), N(season+area)	10	3.60	0.03
S(.), γ″(0), γ′(1), p(season), N(season+area)	12	4.05	0.02
S(sex), γ″(0), γ′(1), p(.), c(.), N(season+area)	10	4.09	0.02
S(.), γ″(.), γ′(.), p(.), N(season+area)	10	4.23	0.02
S(area), γ″(.)=γ′, p(.), N(season+area)	10	4.39	0.02
S(area), γ″(0), γ′ (1), p(.), c(.), N(season+area)	10	4.84	0.02
S(sex), γ″(.)=γ′, p(area) N(season+area)	11	4.85	0.02
S(sex), γ″(.)=γ′, p(.), c(.) N(season+area)	11	5.06	0.01
S(.), γ″(0), γ′(1), p(area), c(area), N(season+area)	11	5.28	0.01
S(.), γ″(.), γ′(.), p(area), N(season+area)	11	5.50	0.01
S(.), γ″(.), γ′(.), p(.), c(.), N(season+area)	11	5.55	0.01
S(area), γ″(.)=γ′, p(.), c(.), N(season+area)	11	5.85	0.01
S(sex), γ″(0), γ′(1), p(area), N(season+area)	13	5.88	0.01
S(sex), γ″(.), γ′(.), p(.), N(season+area)	11	5.93	0.01
S(area), γ″(0), γ′(1), p(season), N(season+area)	13	6.69	0.01
S(area), γ″(.), γ′(.), p(.), N(season+area)	11	6.77	0.01
S(sex), γ″(0), γ′(1), p(area), c(area), N(season+area)	12	7.11	0.01
S(sex), γ″(.), γ′(.), p(.), c(.), N(season+area)	12	7.29	0.00
S(sex), γ″(.), γ′(.), p(area), N(season+area)	12	7.37	0.00
S(area),γ″(0), γ′(1), p(area), c(area), N(season+area)	12	7.87	0.00
S(area), γ″(.), γ′ (.), p(.), N(season+area)	12	8.14	0.00
S(sex), γ″(.)=γ′, p(area), c(area), N(season+area)	13	8.38	0.00

**Table A2:** Model‐averaged estimates of seasonal and overall average population size ($\hat{N}$) and density (leopards per 100 km^2^) calculated using mean maximum distance moved (MMDM). Using a MMDM buffer, the effectively sampled areas were estimated as 381.64 and 358.58 km^2^ for the WSA and ESA, respectively. As discussed in the text, the proportional difference between average WSA and ESA density estimates remains the same regardless of whether HMMDM or MMDM is used to calculate effectively sampled areas (Table 4)

Season	Number Captured	$\hat{N}$	SE	95% LCL‐UCL	Density	95% LCL‐UCL Density
CD 2013, ESA	8	12.53	5.35	8.45–53.85	3.49	2.36–15.02
HD 2013, ESA	5	8.67	4.37	5.36–42.72	2.42	1.49–11.91
CD 2014, ESA	5	8.67	4.37	5.36–42.72	2.42	1.49–11.91
HD 2014, ESA	7	12.11	5.78	7.56–53.91	3.38	2.11–15.03
CD 2012, WSA	16	22.29	5.91	17.00–55.65	5.84	4.45–14.58
CD 2013, WSA	10	18.21	5.88	12.02–43.39	4.77	3.15–11.37
HD 2013, WSA	9	15.71	5.20	10.47–39.66	4.12	2.74–10.39
CD 2014, WSA	9	15.71	5.20	10.47–39.66	4.12	2.74–10.39
HD 2014, WSA	12	21.35	6.44	14.42–48.04	5.59	3.78–12.59
Average, ESA	–	10.50	1.33	7.90–13.10	2.93	2.20–3.65
Average, WSA	–	18.66	1.10	16.50–20.81	4.89	4.32–5.45
